# The Role of PKGIα and AMPK Signaling Interplay in the Regulation of Albumin Permeability in Cultured Rat Podocytes

**DOI:** 10.3390/ijms24043952

**Published:** 2023-02-16

**Authors:** Patrycja Rachubik, Dorota Rogacka, Irena Audzeyenka, Maria Szrejder, Anna Topolewska, Michał Rychłowski, Agnieszka Piwkowska

**Affiliations:** 1 Laboratory of Molecular and Cellular Nephrology, Mossakowski Medical Research Institute, Polish Academy of Sciences, Wita Stwosza 63 St., 80-308 Gdansk, Poland; 2 Faculty of Chemistry, University of Gdansk, Wita Stwosza 63 St., 80-308 Gdansk, Poland; 3 Intercollegiate Faculty of Biotechnology, University of Gdansk, Medical University of Gdansk, Abrahama 58 St., 80-307 Gdansk, Poland

**Keywords:** AMP-activated protein kinase, actin cytoskeleton, albumin permeability, contractile apparatus, podocyte, protein kinase G type Iα

## Abstract

The permeability of the glomerular filtration barrier (GFB) is mainly regulated by podocytes and their foot processes. Protein kinase G type Iα (PKGIα) and adenosine monophosphate-dependent kinase (AMPK) affect the contractile apparatus of podocytes and influence the permeability of the GFB. Therefore, we studied the interplay between PKGIα and AMPK in cultured rat podocytes. The glomerular permeability to albumin and transmembrane FITC-albumin flux decreased in the presence of AMPK activators and increased in the presence of PKG activators. The knockdown of PKGIα or AMPK with small-interfering RNA (siRNA) revealed a mutual interaction between PKGIα and AMPK and influenced podocyte permeability to albumin. Moreover, PKGIα siRNA activated the AMPK-dependent signaling pathway. AMPKα2 siRNA increased basal levels of phosphorylated myosin phosphate target subunit 1 and decreased the phosphorylation of myosin light chain 2. Podocytes that were treated with AMPK or PKG activators were characterized by the different organization of actin filaments within the cell. Our findings suggest that mutual interactions between PKGIα and AMPKα2 regulate the contractile apparatus and permeability of the podocyte monolayer to albumin. Understanding this newly identified molecular mechanism in podocytes provides further insights into the pathogenesis of glomerular disease and novel therapeutic targets for glomerulopathies.

## 1. Introduction

The glomerular filtration barrier (GFB) is responsible for the ultrafiltration of blood plasma that flows through successive layers of the GFB. It is composed of fenestrated endothelial cells, the glomerular basement membrane, and slit diaphragms (SDs), which form between neighboring foot processes (FPs) of podocytes [1]. Podocytes consist of the cell body that floats above the glomerular capillary, major processes, and highly dynamic FPs that attach podocytes to capillaries [2,3]. The complex structure of podocytes is determined by a highly organized actin cytoskeleton [4]. Actin reorganization and alterations of albumin permeability in the podocyte filtration monolayer are closely related to the activity of cyclic guanosine monophosphate (cGMP)-dependent protein kinase G type Iα (PKGIα).

PKGI is a serine/threonine kinase homodimer that regulates the relaxation of the contractile apparatus [5]. PKGI exists in two isoforms: PKGIα and PKGIβ [6]. The classic activation of the enzyme is associated with the binding of cGMP to binding sites of the kinase [7]. An alternative mechanism of PKGIα activation is based on enzyme dimerization, in which a disulfide bond forms between adjacent Cys42 residues in the PKGIα homodimer complex [8] and impairs its activation through the classic cGMP-dependent pathway [9]. Our recent studies have shown that insulin [10], hydrogen peroxide (H_2_O_2_) [11], and high glucose (HG) concentrations [12] mediated the relaxation of the podocyte contractile apparatus via the activation of the PKGIα-dependent signaling pathway. The activated PKGIα triggers the activation of myosin light chain phosphatase (MLCP) through the phosphorylation of its regulatory subunit, myosin phosphate target subunit 1 (MYPT1), at Ser695, which subsequently leads to a decrease in the phosphorylation levels of myosin light chain 2 (MLC2) [10,13,14]. PKGIα signaling to the contractile apparatus triggers actin cytoskeleton reorganization, thereby increasing albumin permeability across the podocyte monolayer [10,11]. Moreover, previous studies reported that the significant augmentation of PKGIα protein expression in hyperinsulinemic and insulin-resistant Zucker rats was associated with an increase in glomerulus permeability to albumin and albuminuria [10]. This evidence suggests that the insulin- and HG-dependent oxidative activation of PKGIα causes the relaxation of the contractile apparatus, which may lead to FP effacement, impairments in podocyte function, and disturbances in GFB permeability.

The proper function of podocytes depends on the energy state of the cell, which is controlled by AMP-activated protein kinase (AMPK). The enzyme consists of three subunits: the catalytic α subunit and regulatory β and γ subunits [15]. The catalytic subunit of AMPKα exists in two isoforms: α1 and α2 [16]. AMPK is activated by AMP, which allosterically activates the enzyme by binding to the regulatory AMPKγ subunit. This allows upstream liver kinase 1 (LKB1) to phosphorylate AMPKα at Thr172, which increases AMPK activity nearly 100-fold [17,18]. Alternative mechanisms of AMPK activation are associated with calcium signaling [19,20,21] and the lysosomal LKB1/Axin/v-adenosine triphosphatase (ATPase)/Ragulator complex [22]. AMPK is crucial in the regulation of glucose and fatty acid metabolism, and modulates cell polarity, permeability, and cytoskeleton reorganization [23,24,25]. In vascular smooth muscle cells, AMPK regulates vasodilatation through the remodeling of the contractile apparatus [25]. The opposite effect was observed in epithelial cells, where AMPK stimulation increased MLC phosphorylation likely in a Rho-kinase-dependent manner [26]. A decrease in both AMPK phosphorylation and activity is associated with kidney dysfunction, especially in diabetes, which suggests a significant role for AMPK in the development of diabetic nephropathy. Our previous studies confirmed that HG significantly decreased AMPK phosphorylation levels and influenced the positive effect of insulin on glucose uptake, leading to the development of insulin resistance in podocytes [27,28]. Metformin (MTF), commonly used as an anti-diabetic drug and AMPK activator, restored both AMPK phosphorylation levels and podocyte sensitivity to insulin under conditions that mimicked diabetes [29], suggesting the key role of AMPK in the regulation of glucose uptake in podocytes. Moreover, glomeruli that were isolated from diabetic rats were characterized by a reduction in the expression of both isoforms, AMPKα1 and AMPKα2, which was associated with kidney dysfunction. An infusion of MTF in the abdominal aorta in diabetic rats led to a decrease in the glomerular filtration rate [29], which may result from the AMPK activation.

Unfortunately, knowledge of the regulatory role of AMPK in actin cytoskeleton organization in podocytes is limited. A small body of evidence describes AMPK activity in the context of GFB permeability. Our previous studies showed that AMPK affected the GFB and podocyte actin dynamics through the stimulation of Rac1 signaling, which belongs to the Rho family of guanosine triphosphatases (GTPases) [21,29]. The inhibition of AMPK activity by compound C and HG promoted the remodeling of actin filaments, which was associated with an increase in albumin permeability across the podocyte monolayer and glomeruli that were isolated from diabetic rats [29]. The preincubation of podocytes with the nonspecific AMPK activator MTF attenuated the effect of HG on the actin filament network and permeability to albumin [29]. These findings suggest a relationship between AMPK activity, actin reorganization, and changes in filtration barrier permeability to albumin. However, the exact function of AMPK in intracellular signaling to the actin cytoskeleton in podocytes is still poorly understood.

Both PKGIα and AMPK regulate filtration barrier permeability in an antagonistic manner. In the present study, we investigated whether the interplay between PKGIα and AMPK signaling in cultured rat podocytes influences filtration barrier permeability. Our results identified a potentially important new mechanism that may be injurious to podocytes in diabetes and affect filtration barrier permeability. Moreover, understanding the interplay between PKGIα and AMPK signaling in podocytes will provide further insights into glomerular disease pathogenesis and novel therapeutic targets for glomerulopathies.

## 2. Results

### 2.1. Role of PKGIα and AMPK in the Regulation of Filtration Barrier Permeability

**Figure 1:**
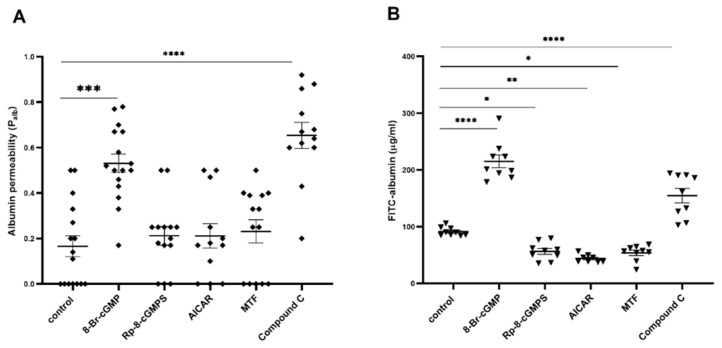
Opposing regulation of filtration barrier permeability by PKGIα and AMPK. Glomerular albumin permeability (P_alb_) (**A**) was evaluated in isolated glomeruli that were exposed to an oncotic gradient, and transepithelial permeability to albumin was assessed by measuring the diffusion of FITC-labeled BSA across the podocyte monolayer (**B**) in the presence of the PKG activator, 8-Br-cGMP (100 μM, 5 min) or the PKG inhibitor Rp-8-Br-cGMPS (100 μM, 20 min) and AMPK activators MTF (2 mM, 30 min for P_alb_ or 2 h for FITC-albumin) and AICAR (100 μM, 20 min for P_alb_ or 2 h for FITC-albumin) or AMPK inhibitor compound C (100 μM, 20 min for P_alb_ or 2 h for FITC-albumin). The data were analyzed using Dunn’s test (non-normal data distribution) or Tukey’s test (normal data distribution), and are expressed as the mean ± SEM (12–16 glomeruli from four rats and 4–6 experiments for podocytes). (**A**): *** *p* < 0.001, **** *p* < 0.0001, compared with control (Dunn’s test). (**B**): * *p* < 0.05, ** *p* < 0.01, **** *p* < 0.0001, compared with control (Tukey’s test).

In the presence of the PKG activator 8-bromoguanosine 3′,5′-cyclic monophosphate (8-Br-cGMP; 100 μM, 5 min), significant increases in the albumin permeability (P_alb_) of isolated glomeruli (0.166 ± 0.046 vs. 0.531 ± 0.041, *n* = 16, *p* < 0.0001; Figure 1A) and podocyte monolayer permeability (from 91.370 ± 2.551 to 215 ± 11.420 μg/mL, *n* = 9, *p* < 0.0001; Figure 1B) were observed. Podocyte albumin permeability was partially inhibited by the PKG inhibitor Rp-8-Br-cGMPS (100 μM, 20 min), which resulted in FITC albumin permeability of 56.520 ± 5.148 μg/mL (*n* = 9, *p* < 0.05). The AMPK activators 5-aminoimidazole-4-carbox-amide-1-β-D-ribofuranoside (AICAR; 100 μM, 2 h) and MTF (2 mM, 2 h) markedly diminished FITC-albumin permeability to the podocyte monolayer by 52% (91.370 ± 2.551 vs. 43.950 ± 2.415 μg/mL, *n* = 9, *p* < 0.01) and 41% (91.370 ± 2.551 vs. 53.700 ± 4.574 μg/mL, *n* = 9, *p* < 0.05), respectively. A reduction in glomerular P_alb_ was not observed in response to AICAR (100 μM, 20 min) or MTF (2 mM, 30 min), likely because of the greater complexity of the glomerular structure. This limitation should be considered, especially when treatments may influence the contractile state of mesangial cells, which might impact the glomerular volume. However, albumin permeability increased substantially in both the isolated glomeruli (from 0.17 ± 0.05 to 0.65 ± 0.06 μg/mL, *n* = 12, *p* < 0.0001) and podocytes (from 91.370 ± 2.551 to 154.700 ± 12.600 μg/mL, *n* = 9, *p* < 0.0001) that were exposed to the AMPK inhibitor compound C (100 μM, 20 min for P_alb_ or 2 h for FITC-albumin). These results suggest that filtration barrier permeability is regulated by PKGIα and AMPK signaling in an antagonistic manner.

### 2.2. Downregulation of AMPK and PKGIα Affects Albumin Permeability across the Podocyte Monolayer

**Figure 2:**
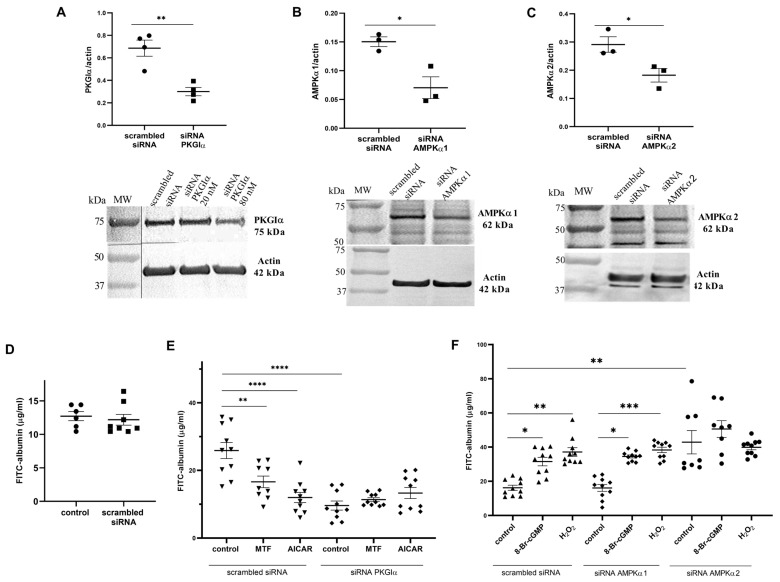
Opposite effects of PKGIα and AMPKα on albumin permeability across the podocyte monolayer. (**A**–**C**) Effects of PKGIα (**A**), AMPKα1 (**B**), and AMPKα2 (**C**) gene-silencing on protein expression in cultured rat podocytes; (**D**) Effect of scrambled siRNA on podocyte albumin permeability; (**E**) Effect of PKGIα siRNA on podocyte albumin permeability in the presence of the AMPK activators MTF (2 mM, 2 h) and AICAR (100 μM, 2 h); (**F**) Effect of AMPKα1 and AMPKα2 gene-silencing on podocyte permeability in the presence of the PKGI activators 8-Br-cGMP (100 μM, 5 min) and H_2_O_2_ (100 μM, 5 min). The data were analyzed using unpaired *t*-test, Dunn’s test, or Tukey’s test and are expressed as the mean ± SEM. *n* = 3–12. * *p* < 0.05, ** *p* < 0.01, *** *p* < 0.001, **** *p* < 0.0001, compared with appropriate siRNA control.

Previous studies showed that PKGIα activation is associated with an increase in the permeability of the podocyte monolayer to albumin, whereas AMPK activity is linked to the opposite effect. To determine whether changes in podocyte permeability to albumin arise from crosstalk between AMPKα and PKGIα, podocytes were transfected with siRNA that targeted PKGIα, AMPKα1, or AMPKα2. To assess the efficacy of siRNA transfection, the expression of PKGIα, AMPKα1, and AMPKα2 proteins was determined in podocytes that were transfected with PKGIα, AMPKα1, or AMPKα2 siRNA. PKGIα siRNA-treated cells exhibited significantly (56%) lower levels of PKGIα protein (from 0.687 ± 0.072 to 0.300 ± 0.037, *n* = 4, *p* < 0.01; Figure 2A). The downregulation of AMPKα1 or AMPKα2 gene expression resulted in a 53% decrease in protein levels (from 0.150 ± 0.008 to 0.070 ± 0.019 *n* = 3, *p* < 0.05) for AMPKα1 (Figure 2B) and a 37% decrease in protein levels (from 0.291 ± 0.027 to 0.182 ± 0.024, *n* = 3, *p* < 0.05) for AMPKα2 (Figure 2C). Transfection with scrambled siRNA did not alter podocyte permeability to albumin (Figure 2D).

Subsequently, to determine if PKGIα is involved in AMPK-dependent regulation of podocyte permeability to albumin, podocytes were transfected with siRNA that targeted PKGIα and then, 48 h after transfection, incubated with MTF or AICAR. We found that both MTF and AICAR significantly decreased permeability of the control podocyte monolayer to albumin by 36% (25.876 ± 2.376 vs. 16.619 ± 1.718 μg/mL, *n* = 10, *p* < 0.01) and 53.6% (25.876 ± 2.376 vs. 11.995 ± 1.502 μg/mL, *n* = 10, *p* < 0.0001; Figure 2E), respectively. The silencing of PKGIα expression by siRNA resulted in a 62.8% reduction in FITC-albumin permeability across the podocyte monolayer (25.876 ± 2.376 vs. 9.616 ± 1.340 μg/mL, *n* = 10, *p* < 0.0001; Figure 2E). Neither MTF nor AICAR influenced podocyte permeability to albumin in cells that were transfected with PKGIα siRNA (Figure 2E). Next, the regulatory effect of AMPKα on the PKGIα-dependent modulation of podocyte monolayer permeability to FITC-albumin was evaluated. As shown in Figure 2F, a significant increase in FITC-albumin permeability was observed in podocytes that were treated with 8-Br-cGMP (from 16.237 ± 1.494 to 31.541 ± 2.481 μg/mL, *n* = 10, *p* < 0.01) and H_2_O_2_ (from 16.237 ± 1.494 to 37.112 ± 2.549 μg/mL, *n* = 10, *p* < 0.0001). Analogous experiments were performed with the siRNA-mediated knockdown of AMPKα1, and similar results were obtained (Figure 2F). However, AMPKα2 siRNA caused a 2.6-fold increase in permeability to FITC-albumin across the podocyte monolayer (from 16.237 ± 1.494 to 42.86 ± 6.835 μg/mL, *n* = 8–10, *p* < 0.0001; Figure 2F). These results suggest that the PKGIα and AMPKα2 isoforms are involved in the antagonistic regulation of albumin permeability across the podocyte monolayer. Disturbances in AMPKα2 activity may augment filtration barrier permeability, at least partially, in a PKGIα-dependent manner.

### 2.3. PKGIα Affects AMPK Activity in Podocytes

**Figure 3:**
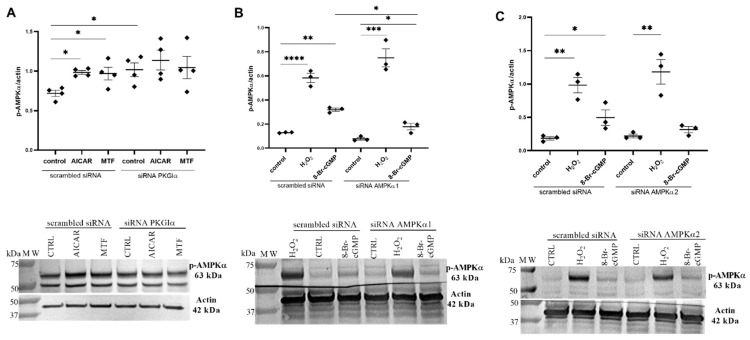
Interplay between PKGIα and AMPK affects AMPK activity. (**A**) Influence of PKGIα siRNA on AMPK phosphorylation in the presence of the AMPK activators MTF (2 mM, 2 h) and AICAR (100 μM, 2 h); (**B**,**C**) Effect of AMPKα1 (**B**) and AMPKα2 (**C**) siRNAs on AMPK phosphorylation in the presence of the PKGI activators 8-Br-cGMP (100 μM, 5 min) and H_2_O_2_ (100 μM, 5 min). The data were analyzed using Dunnett’s test, Sidak’s test, or unpaired *t*-test and are expressed as the mean ± SEM. *n* = 3–4. * *p* < 0.05, ** *p* < 0.01, *** *p* < 0.001, **** *p* < 0.0001, compared with appropriate siRNA control.

Next, to investigate the effect of PKGIα on AMPKα phosphorylation, the PKGIα expression was selectively knocked down, and podocytes were incubated with AICAR or MTF. Consistent with numerous previous studies, Figure 3A shows that AMPKα phosphorylation levels increased by 37% (0.718 ± 0.039 vs. 0.984 ± 0.024, *n* = 4, *p* < 0.01) for AICAR and by 35% (0.718 ± 0.039 vs. 0.968 ± 0.080, *n* = 4, *p* < 0.05) for MTF. Unexpectedly, the siRNA-mediated knockdown of PKGIα increased the AMPKα phosphorylation levels by 42% (0.718 ± 0.039 vs. 1.017 ± 0.087, *n* = 4, *p* < 0.05; Figure 3A) in control cells. We next tested whether PKGIα activation influences AMPKα phosphorylation. 8-Br-cGMP and H_2_O_2_ were used as PKGIα activators. 8-Br-cGMP is responsible for the classical activation of PKGIα, whereas H_2_O_2_ induces the non-canonical activation of the enzyme, called oxidative activation, involving the formation of an intermolecular disulfide. The incubation of podocytes with either H_2_O_2_ or 8-Br-cGMP increased the phosphorylation state of AMPKα 4.3-fold (0.135 ± 0.006 vs. 0.583 ± 0.038, *n* = 3–4, *p* < 0.0001; Figure 3B) and 2.4-fold (0.135 ± 0.006 vs. 0.319 ± 0.013, *n* = 3–4, *p* < 0.01; Figure 3B), respectively. The siRNA-mediated silencing of AMPKα1 (Figure 3B) or AMPKα2 (Figure 3C) had no influence on the H_2_O_2_-dependent increase in AMPKα phosphorylation. However, the effect of 8-Br-cGMP on the level of AMPKα phosphorylation was substantially decreased by AMPKα1 siRNA (Figure 3B). As shown in Figure 3C, AMPKα phosphorylation did not decrease in podocytes that were transfected with AMPKα2 siRNA alone, but the positive effect of 8-Br-cGMP on the AMPKα phosphorylation state was slightly reduced by AMPKα2 siRNA. These results suggest that PKGIα’s interaction with AMPK regulates the activity of both AMPK isoforms (α1 and α2). However, the AMPKα2 isoform may be mainly involved in signal transduction, which is associated with the classic activation of PKGIα by cGMP.

### 2.4. AMPKα Interacts with PKGIα in Cultured Rat Podocytes

**Figure 4:**
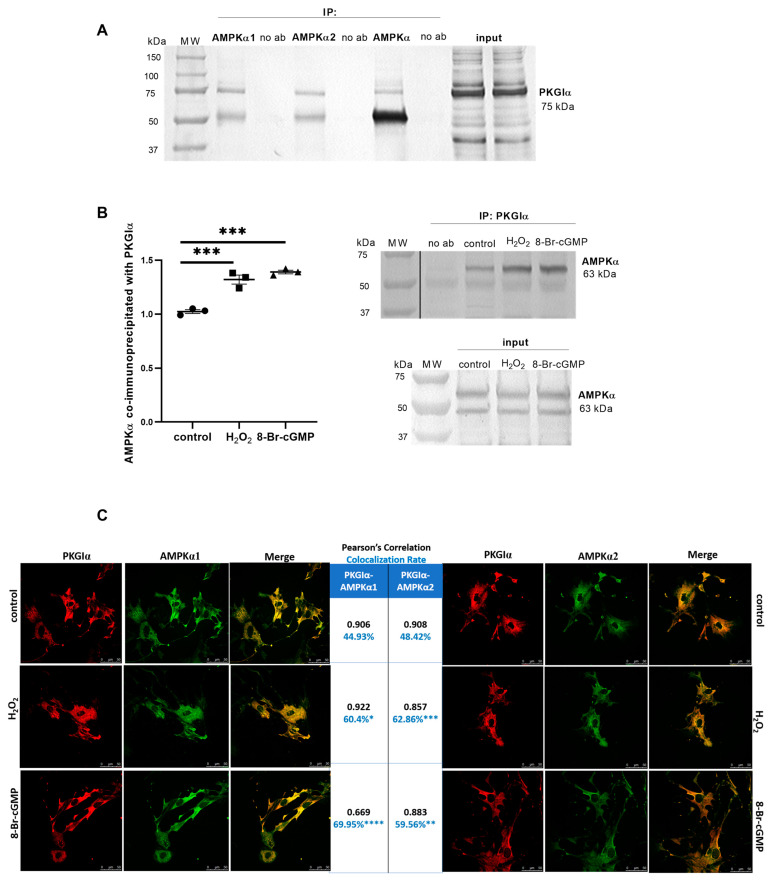
PKGIα and AMPKα form a complex in cultured rat podocytes. (**A**) Immunoblots that show that PKGIα is associated with immunoprecipitated AMPKα1 and AMPKα2 subunits in podocyte extracts; (**B**) Densitometry analysis of coimmunoprecipitation immunoblots comparing coimmunoprecipitated protein to total protein (input). H_2_O_2_ and 8-Br-cGMP (100 μM, 5 min) increased the amount of AMPKα that co-immunoprecipitated with PKGIα (*n* = 3, *** *p* < 0.001, Dunnett’s test). Right panel: representative co-immunoprecipitation immunoblots; (**C**) Podocytes were seeded on coverslips, treated with H_2_O_2_ or 8-Br-cGMP (100 μM, 5 min), and then incubated with anti-PKGIα and anti-AMPKα1 or anti-AMPKα2 antibodies as indicated. Pixel intensities were quantified. The results are reported as Pearson correlation coefficients and colocalization rates (%). The quantitative analysis of protein colocalization was performed with LAS AF 3.3.0 software (*n* = 11, * *p* < 0.05, ** *p* < 0.01, *** *p* < 0.001, **** *p* < 0.0001; AMPKα1: Dunn’s test, AMPKα2: Dunnett’s test). Bar scale = 50 μm. Objective magnification: 63×.

To further confirm the interaction between PKGIα and AMPKα, we conducted the co-immunoprecipitation of these two proteins, showing that PKGIα formed a complex with both AMPKα1 and AMPKα2 (Figure 4A). In a reverse procedure, AMPKα formed a complex with PKGIα (Figure 4B). The treatment of podocytes with H*_2_*O*_2_* or 8-Br-cGMP increased the amount of AMPKα that co-immunoprecipitated with PKGIα by 29% (1.023 ± 0.017 vs. 1.320 ± 0.042, *n* = 3, *p* < 0.001) and 36% (1.023 ± 0.017 vs. 1.390 ± 0.017, *n* = 3, *p* < 0.001), respectively (Figure 4B).

Moreover, confocal immunofluorescence images of podocytes confirmed the very strong positive correlation of PKGIα with AMPKα1 (0.906 ± 0.008, *n* = 11) and showed an increase in the colocalization of PKGIα with AMPKα1 by 15.5% (44.93% ± 4.22% vs. 60.40% ± 2.49%, *n* = 11, *p* < 0.05) and 25% (44.93% ± 4.22% vs. 69.95% ± 2.48%, *n* = 11, *p* < 0.0001) for H_2_O_2_ and 8-Br-cGMP, respectively (Figure 4C). PKGIα also very strongly positively correlated with AMPKα2 (0.908 ± 0.008, *n* = 11). The incubation of podocytes with H_2_O_2_ or 8-Br-cGMP increased the colocalization of PKGIα with AMPKα2 by 14.4% (48.42% ± 2.37% vs. 62.86% ± 2.64%, *n* = 11, *p* < 0.001) and 11.1% (48.42% ± 2.37% vs. 59.56% ± 1.76%, *n* = 11, *p* < 0.01), respectively (Figure 4C). However, there were no visible changes in the cell distribution of these two kinases in podocytes.

### 2.5. PKG and AMPK Modulators Affect Nucleotide Concentrations in Cultured Rat Podocytes

**Figure 5:**
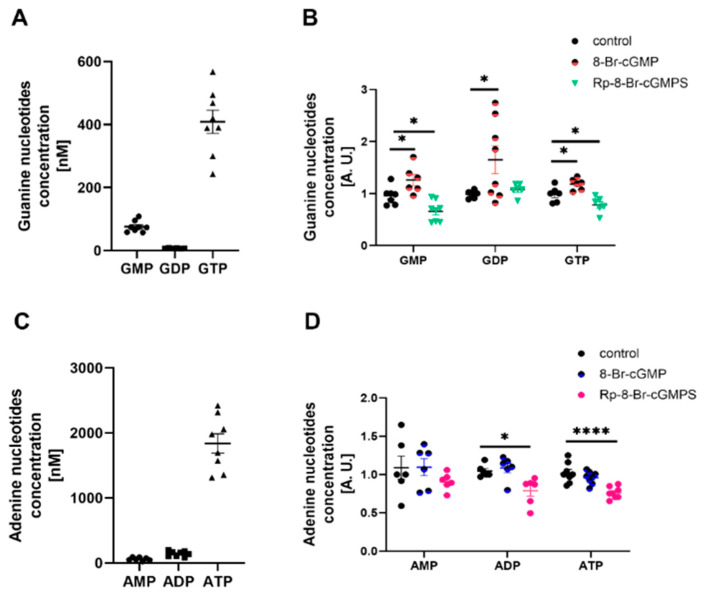
Effect of PKG modulators on the energy state of podocytes. (**A**,**C**) Guanine (**A**) and adenine (**C**) nucleotide levels in cultured rat podocytes; (**B**,**D**) Effect of 8-Br-cGMP (PKG activator, 100 μM, 5 min) and Rp-8-Br-cGMPS (PKG inhibitor, 100 μM, 20 min) on concentrations of guanine (**B**) and adenine (**D**) nucleotides in cultured podocytes. The data were analyzed using Dunnett’s test and are expressed as the mean ± SEM. *n* = 6–8. * *p* < 0.05, **** *p* < 0.0001, compared with appropriate control.

**Figure 6:**
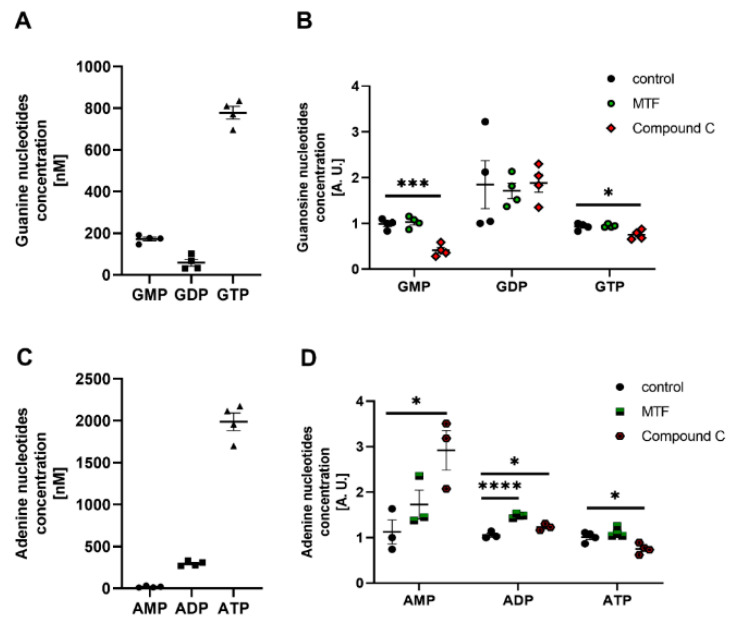
Effect of AMPK modulators on the energy state of podocytes. (**A**,**C**) Guanine (**A**) and adenine (**C**) nucleotide levels in cultured rat podocytes; (**B**,**D**) Effect of MTF (AMPK activator, 2 mM, 2 h) and compound C (AMPK inhibitor, 100 μM, 2 h) on concentrations of guanine (**B**) and adenine (**D**) nucleotides in cultured podocytes. The data were analyzed using Dunnett’s test and are expressed as the mean ± SEM. *n* = 3–4. * *p* < 0.05, *** *p* < 0.001, **** *p* < 0.0001, compared with appropriate control.

The classic activation of PKG and AMPK is based on changes in cGMP and ATP concentrations, respectively. Therefore, we investigated whether PKG and AMPK modulators alter the concentration of adenine (AMP, adenosine diphosphate (ADP), and ATP) and guanine (GMP, guanosine diphosphate (GDP), and GTP) nucleotides. Podocytes can produce energy, reflected by the high intracellular concentrations of GTP (Figure 5A) and ATP (Figure 5C). The use of 8-Br-cGMP increased GMP, GDP, and GTP concentrations by 31%, 67%, and 20%, respectively (Figure 5B). However, it did not affect the concentrations of adenine nucleotides (Figure 5D). Podocytes that were treated with Rp-8-Br-cGMPS were characterized by lower amounts of GMP and GTP (Figure 5B), and ADP and ATP (Figure 5D). In a reverse procedure, AMPK modulators were administered. Subsequently, the effect of an AMPK inhibitor, compound C, on nucleotide concentration in podocytes was determined. Compound C significantly reduced GMP (58%), GTP (20%), ADP (17%), and ATP (26%) concentrations but increased AMP concentrations from 1.127 ± 0.264 to 2.922 ± 0.434 (*n* = 3, *p* < 0.05; Figure 6B,D). MTF did not affect the concentration of guanine or adenine nucleotides, with the exception of ADP (Figure 6B,D).

Overall, these experiments suggest that PKG and AMPK modulators affect nucleotide levels, which may subsequently impact PKGIα and AMPK activity in podocytes.

### 2.6. PKGIα and AMPK Mutually Regulate the Contractile Apparatus in Podocytes

**Figure 7:**
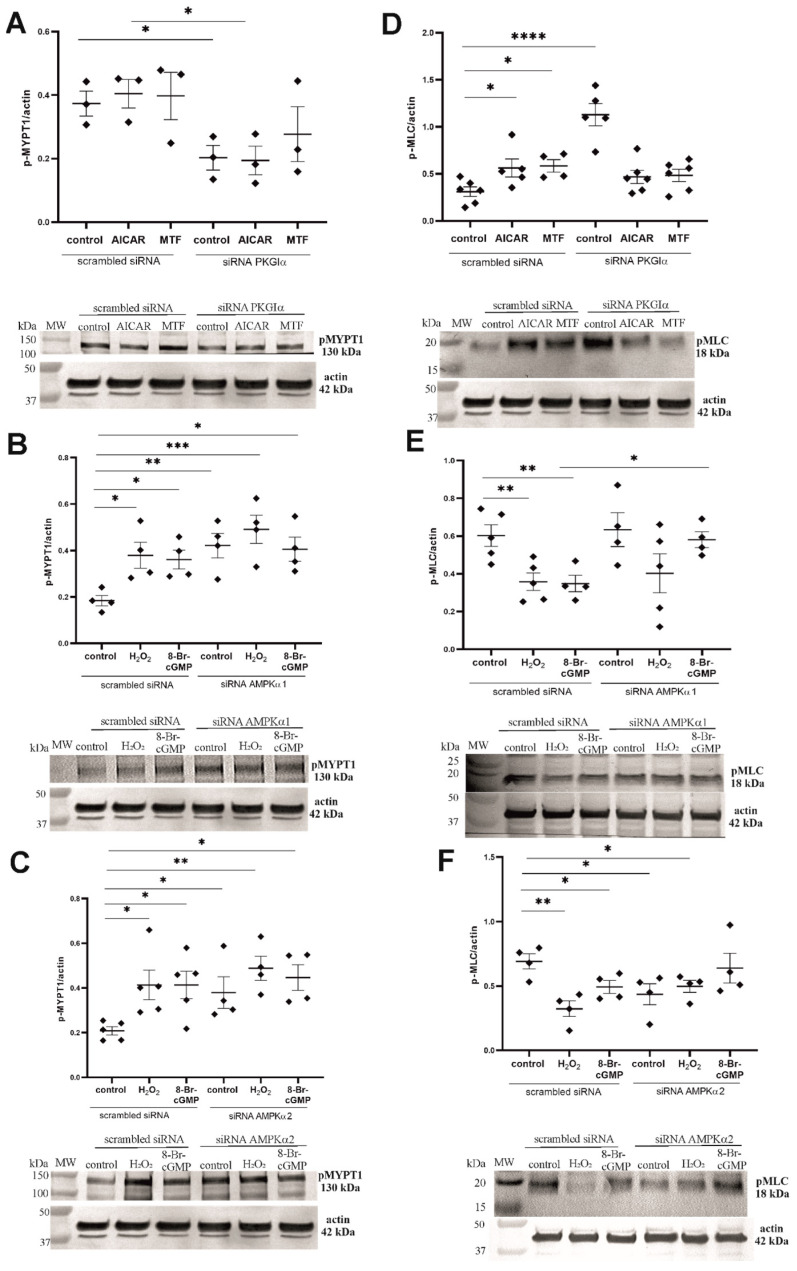
PKGIα and AMPK mutually regulate the contractile apparatus in cultured rat podocytes. (**A**,**B**) Effect of PKGIα gene-silencing on MYPT1 (**A**) and MLC (**B**) phosphorylation in the presence of the AMPK activators MTF (2 mM, 2 h) and AICAR (100 μM, 2 h); (**C**–**F**) Effect of AMPKα1 (**C**,**D**) and AMPKα2 (**E**,**F**) gene-silencing on MYPT1 and MLC phosphorylation in the presence of the PKGI activators 8-Br-cGMP (100 μM, 5 min) and H_2_O_2_ (100 μM, 5 min). The data were analyzed using Sidak’s test, Dunnett’s test, or unpaired *t*-test and are expressed as the mean ± SEM. *n* = 3–6. * *p* < 0.05, ** *p* < 0.01, *** *p* < 0.001, **** *p* < 0.0001, compared with appropriate siRNA control.

Considering that PKGIα regulates MLC activity through MYPT1 phosphorylation at Ser695 [10] and that AMPK negatively regulates MYP1 phosphorylation at Thr696 [30], we hypothesized that the interplay between PKGIα and AMPK affects the contractile apparatus in podocytes. Neither AICAR nor MTF influenced the MYPT1 phosphorylation state (Figure 7A). However, PKGIα silencing decreased the level of phosphorylated MYPT1 by 45.7% (0.373 ± 0.039 vs. 0.203 ± 0.039, *n* = 3, *p* < 0.05) in control cells and by 52% (0.404 ± 0.045 vs. 0.194 ± 0.045 *n* = 3, *p* < 0.05) in podocytes that were treated with AICAR (Figure 7A), suggesting the PKGIα-dependent regulation of the MYPT1 activity. Consistent with numerous previous studies, Figure 7B shows that PKGIα activators markedly increased the level of MYPT1 phosphorylation by 105% (0.184 ± 0.022 vs. 0.379 ± 0.056, *n* = 4, *p* < 0.05) for H_2_O_2_ and by 96% (0.184 ± 0.022 vs. 0.361 ± 0.041, *n* = 4, *p* < 0.05) for 8-Br-cGMP. AMPKα1-directed siRNA significantly increased the amount of phosphorylated MYPT1 (from 0.184 ± 0.022 to 0.421 ± 0.053, *n* = 4, *p* < 0.01; Figure 7B). Similar increases were found with AMPKα2 siRNA (0.208 ± 0.018 vs. 0.379 ± 0.054, *n* = 4–5, *p* < 0.05; Figure 7C). These results suggest that both AMPKα isoforms play a role in regulating the MYPT1 phosphorylation state.

Next, we tested whether the interplay between PKGIα and AMPK affects the MLC phosphorylation state. The administration of AMPK activators increased MLC phosphorylation by 81% (0.310 ± 0.051 vs. 0.562 ± 0.095, *n* = 5–6, *p* < 0.05) for AICAR and by 88% (0.310 ± 0.051 vs. 0.584 ± 0.066, *n* = 4–6, *p* < 0.05) for MTF (Figure 7D). MLC phosphorylation significantly increased from 0.310 ± 0.051 to 1.129 ± 0.118 (*n* = 5–6, *p* < 0.0001) in podocytes that were transfected with PKGIα siRNA, whereas no changes in MLC phosphorylation were found in podocytes that were incubated with AICAR or MTF (Figure 7D). The reduction in AMPKα1 expression by siRNA did not influence the MLC phosphorylation state (Figure 7E) but attenuated the effect of both 8-Br-cGMP and H_2_O_2_ on it. However, when using siRNA against AMPKα2, a decrease in the basal levels of phosphorylated MLC (0.692 ± 0.059 vs. 0.435 ± 0.081, n = 4, *p* < 0.05; Figure 7F) was observed,

### 2.7. PKGIα and AMPK Affect Actin Cytoskeleton Architecture in an Antagonistic Manner

**Figure 8:**
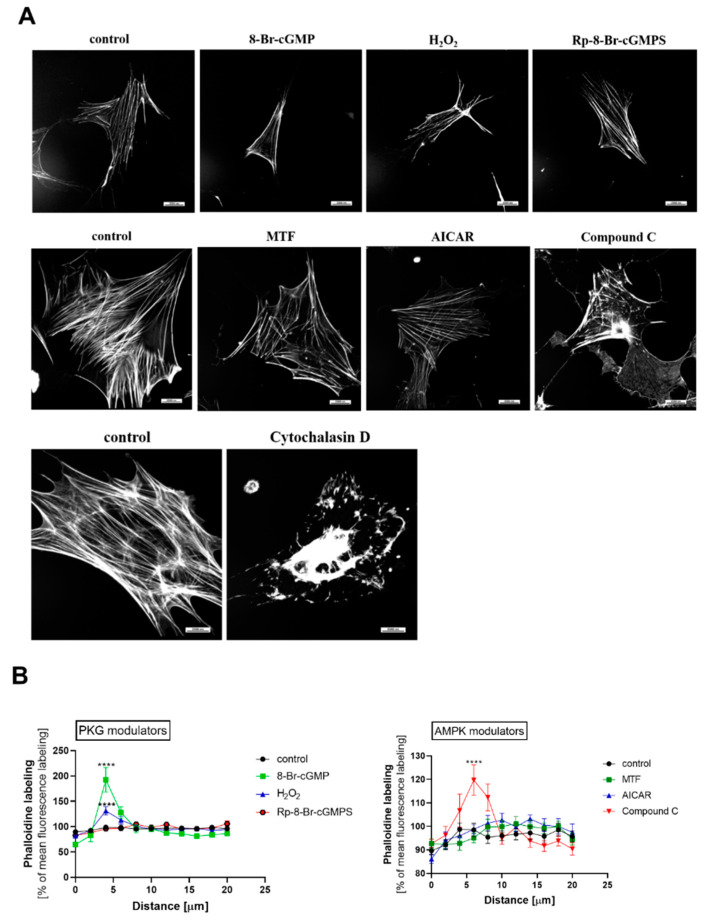
PKGIα and AMPK antagonistically mediate the remodeling of actin filaments in cultured podocytes. Podocytes were seeded on glass coverslips and treated with PKG modulators (8-Br-cGMP, 100 μM, 5 min; H_2_O_2_, 100 μM, 5 min; Rp-8-Br-cGMPS, 100 μM, 20 min), AMPK modulators (MTF, 2 mM, 2 h; AICAR, 100 μM, 2 h; compound C, 100 μM, 2 h), or the actin filament disruptor cytochalasin D as a positive control (10 μM, 30 min). (**A**) The F-actin network was labeled with phalloidin (1:200) and visualized by confocal microscopy; (**B**) Analysis of fluorescence intensity profile (F-actin distribution within the cell was measured as the distance from the basal membrane (0 μm) to the nucleus (20 μm)) using Nikon NIS-Elements software. Scale bar = 25 μm. Objective magnification: 40×. The data were analyzed using Dunnett’s test and are expressed as the mean ± SEM. *n* = 11–17. **** *p* < 0.0001.

Based on our findings that PKGIα and AMPKα2 mutually regulated the phosphorylation states of MYPT1 and MLC, we next investigated whether changes in PKGIα and AMPK activity are associated with actin cytoskeleton reorganization in rat podocytes. Either PKG or AMPK modulators were administered, which considerably influenced actin filament organization. The PKG activators 8-Br-cGMP and H_2_O_2_ and AMPK inhibitor compound C significantly increased F-actin immunostaining near the plasma membrane, whereas the incubation of podocytes with PKG inhibitors or AMPK activators had no effect on actin organization (Figure 8). Cytochalasin D (10 μM, 30 min) was used as a positive control of cytoskeleton disruption (Figure 8A).

## 3. Discussion

Podocytes are contractile cells that dynamically reorganize their actin cytoskeleton to regulate GFB permeability in response to environmental stimuli. In podocytes, PKGIα and AMPK antagonistically regulate filtration barrier permeability through the indirect modulation of actin architecture. Insulin-resistant podocytes are characterized by augmented PKGIα activity and diminished AMPK phosphorylation, resulting in an increase in albumin permeability across the podocyte monolayer and isolated glomeruli [10,29,31]. The inhibition of PKGIα activity or AMPK activation prevented actin cytoskeleton reorganization and decreased albumin permeability across the filtration barrier. Thus, we propose that the interplay between the PKGIα and AMPKα2 activity regulates the contraction apparatus and permeability to albumin in podocytes. The present study revealed a new mechanism in podocytes that may be injurious in diabetes, and alterations of the activity of one of these enzymes may alter filtration barrier permeability.

In the present study, we demonstrated that PKGIα and AMPK antagonistically regulated albumin permeability across the podocyte monolayer and isolated glomeruli (Figure 1). This is consistent with our previous findings, in which the treatment of insulin-resistant podocytes with MTF increased AMPK phosphorylation and decreased permeability to albumin across the podocyte monolayer and diabetic glomeruli [29]. Numerous studies found that AMPK activation improved lung endothelial barrier function by decreasing vascular permeability [32], and reduced both paracellular FITC-dextran permeability across the Caco2-cell monolayer and intestinal permeability to FITC-dextran 40 in vivo [33]. The AMPK inhibitor compound C exerted a negative effect on filtration barrier function (Figure 1B), whereas the increase in filtration barrier permeability was associated with PKGIα activation by insulin [10] or HG [12]. Additionally, hyperinsulinemic and insulin-resistant obese Zucker rats were characterized by the higher expression of the PKGIα protein, polyuria, and albuminuria [10]. Wu et al. found that the hyperpermeability of coronary venules was mediated by the activation of PKG [34]. Studies of heart tissues also confirmed that PKGIα mediated the increase in vascular permeability [35].

Expression levels of AMPKα isoforms differ in various cells. Based on our recent findings that AMPKα1 and AMPKα2 are constitutively expressed in podocytes [27], we studied the effects of these two isoforms on podocyte monolayer permeability to albumin. An increase in albumin permeability across the podocyte monolayer was observed only in podocytes with the knockdown of AMPKα2 expression (Figure 2F), suggesting a regulatory role for this isoform in albumin permeability. Furthermore, the co-immunoprecipitation and immunofluorescence results demonstrated that both AMPKα isoforms interact with PKGIα (Figure 4).

AMPKα1 and AMPKα2 isoforms overlap. In mouse primary proximal tubular cells, α isoforms decrease cell death that is caused by metabolic stress, and α isoforms of AMPK can substitute each other [36]. Mahboubi et al. demonstrated that both AMPKα1 and AMPKα2 isoforms are relevant for cell survival in response to stress [37]. A previous study showed that 8-Br-cGMP increased the degree of AMPK phosphorylation at Thr172 [38]. The present study found that targeting AMPKα1 or AMPKα2 for knockdown did not affect the phosphorylation of the enzyme by the H_2_O_2_ or cGMP analog (Figure 3B, C). To explain this lack of change in the phosphorylation state of AMPK for the podocytes that exhibited the downregulation of AMPKα1 or AMPKα2 expression, we suggest that there is a compensatory mechanism of the intact α isoform in both AMPKα knockdown sets of podocytes. However, the two α isoforms also exhibit unique functions within the cell. A growing body of evidence suggests that different AMPK-dependent cellular effects are determined by the stimulation of the AMPKα1 or AMPKα2 isoform. The AMPKα2 isoform responds to transient receptor potential channel 6-dependent Ca^2+^ signaling, and is involved in the insulin-dependent regulation of glucose uptake in cultured rat podocytes [21]. Szrejder et al. postulated that MTF induced AMPKα1 activation to reduce transient receptor potential channel 6 expression in podocytes that were exposed to HG [29]. However, MTF increases the activity of both AMPKα1 and AMPKα2 isoforms in skeletal muscle cells, where enzyme activation is linked to an increase in glucose uptake [39]. This demonstrates that the action of AMPKα also depends on the type and function of the cell. The stimulation of brain microvascular endothelial cells by vasodilators activates the endothelial nitric oxide synthase/nitric oxide (NO) signaling pathway by the Ca^2+^-dependent stimulation of AMPKα1, leading to acute vascular permeability [24]. Nitric oxide signaling activates the cGMP/PKG signaling pathway and induces vasodilatation through a decrease in intracellular Ca^2+^ concentration [40]. One speculation is that the AMPKα1-dependent activation of the NO/cGMP/PKG signaling pathway might increase vascular permeability in brain endothelial cells. In the present study, transfection with PKGIα siRNA significantly reduced transmembrane albumin flux across the podocyte monolayer to values that were similar to MTF and AICAR. Altogether, these findings suggest that the reciprocal regulation of PKGIα and AMPKα2 activity impacts podocyte permeability to albumin under physiological conditions.

The incubation of ventromedial hypothalamus neurons with the cGMP analog 8-Br-cGMP also increased AMPKα2 phosphorylation [41], suggesting that PKG may influence AMPKα activity. H_2_O_2_ is also known to be implicated in the oxidative activation of PKGIα [11] in podocytes, and increases AMPK phosphorylation through an increase in the intracellular AMP/ATP ratio [42]. AMPK activation leads to a decrease in reactive oxygen species generation by NADPH oxidase [43]. AMPK may regulate PKGIα activity through the inhibition of the NADPH oxidase-dependent production of reactive oxygen species in cultured rat podocytes, but this hypothesis needs to be verified. The present study found that PKGIα affected AMPKα phosphorylation in podocytes. The downregulation of PKGIα expression and PKG activators increased basal levels of phosphorylated AMPKα (Figure 3A). Furthermore, AMPKα1 siRNA partially abolished the positive effect of 8-Br-cGMP on the phosphorylation state of AMPKα (Figure 3A). We also found that H_2_O_2_ and 8-Br-cGMP treatment increased the amount of the AMPKα/PKGIα complex (Figure 4B) and increased the colocalization of PKGIα with AMPKα1 and AMPKα2 without altering the cellular distribution of these proteins in podocytes (Figure 4C). These results are consistent with Ramnanan et al., in which both AMPKα1 protein levels and activity increased in PKG immunoprecipitates from estivated snail foot muscle and hepatopancreas [44]. The H_2_O_2_- and 8-Br-cGMP-dependent activation of PKGIα and both AMPKα isoforms might promote the recruitment of these enzymes to a protein complex, where PKGIα and AMPKα reciprocally modulate each other’s activity and might coordinate the intensity of signaling to appropriate effectors. Increases in the PKGIα and AMPKα interaction in response to specific stimuli may be necessary for the activation of these enzymes. Notably, PKGIα and AMPK activity also depends on GTP and ATP levels; therefore, changes in nucleotide concentrations may modify PKGIα- and AMPK-dependent signaling. We found that cGMP analogs, such as 8-Br-cGMP and Rp-8-Br-cGMPS, exert a minimal effect on GTP and ATP levels (Figure 5), which may result from changes in the local pool of GTP and ATP that are necessary to switch on/off individual signaling pathways.

The podocyte contractile apparatus consists of actin filaments, myosin II, α-actinin-4, synaptopodin, talin, vinculin, and vimentin [45,46]. Cell contraction is based on a direct interaction between myosin and actin filaments. The contractility of the apparatus likely highly depends on the MLC phosphorylation state. Previous studies showed that the insulin- or 8-Br-cGMP-dependent activation of PKGIα increased MYPT1 and decreased MLC phosphorylation, resulting in the reorganization of the actin cytoskeleton in podocytes [47]. In vascular smooth muscle cells, AMPK is also involved in the regulation of cell contractility [25,30], but the exact role of AMPK in regulating the podocyte contractile apparatus is poorly known.

In the present study, we confirmed that siRNA-dependent PKGIα gene-silencing decreased MYPT1 phosphorylation as much as AICAR or MTF (Figure 7A). Moreover, the selective knockdown of PKGIα expression markedly increased MLC phosphorylation, and the effect was stronger than that of the treatment with AMPK activators alone (Figure 7D). However, we did not observe any additive effects of PKGIα siRNA and AMPK activators on the MLC phosphorylation state. This suggests that AMPK may maintain the phosphorylation of MYPT1 and MLC at basal levels through the indirect attenuation of PKGIα activity or inhibition of the interaction between PKGIα and MYPT1, resulting in the protection of the podocyte contractile apparatus against its uncontrolled relaxation. This hypothesis appears to be supported by our findings that siRNA against AMPKα2 markedly increased the basal levels of phosphorylated MYPT1 but decreased phosphorylated MLC to values that were similar to PKG activators (Figure 7C,F). We did not observe any changes in the phosphorylation state of MLC after transfecting podocytes with AMPKα1 siRNA, but the effects of 8-Br-cGMP and H_2_O_2_ on MLC phosphorylation were attenuated in these cells (Figure 7B,E). These results may suggest that AMPKα2 is involved in regulating the MLC phosphorylation state, and crosstalk between PKGIα and AMPKα2 activity may control the contractile apparatus in podocytes.

Changes in MLC phosphorylation appear to correspond to the alterations of the organization of actin filaments in podocytes after the application of PKG and AMPK modulators. 8-Br-cGMP- and H_2_O_2_-treated cells are characterized by the accumulation of actin filaments near the plasma membrane, and a similar effect was observed with the administration of the AMPK inhibitor compound C (Figure 8).

The regulation of actin remodeling is controlled by the concentrations of Ca^2+^ [48,49] and small GTP-binding proteins, such as Rac1, RhoA, and Cdc42c [50]. In podocytes, we found that the insulin-dependent activation of PKGIα increased Rac1 activity, which triggers actin filament reorganization through the filament-severing function of cofilin [51]. Moreover, Rac1-silencing restored basal MLC phosphorylation to control levels and prevented actin remodeling in podocytes that were treated with insulin or 8-Br-cGMP [51]. Experiments on insulin-treated podocytes also showed that the Ca^2+^-dependent activation of AMPKα2 was required to stimulate the Rac1/PAK/cofilin pathway, resulting in actin filament rearrangement [21]. These findings suggest that Rac1-dependent actin remodeling may be at least partially under the control of the PKGIα/AMPKα2 complex.

## 4. Materials and Methods

### 4.1. Preparation and Culture of Rat Podocytes

All experiments were performed in accordance with directive 2010/63/EU for animal experiments, and the protocol was approved by the local ethics committee of the University of Science and Technology, Bydgoszcz, Poland.

Female Wistar rats, weighing 120–140 g, were used for primary podocyte culture as described previously [11]. All experiments were performed using podocytes that were cultured for 12–20 days. Cell phenotypes were confirmed by podocyte-specific antibodies against Wilms tumor-1 protein (Biotrend, Koeln, Germany) and synaptopodin (Progen, Heidelberg, Germany).

### 4.2. Western Blot

To obtain podocyte lysates, the cells were treated with lysis buffer (1% Nonidet P-40, 20 mM Tris, 140 mM NaCl, 2 mM ethylenediaminetetraacetic acid, and 10% glycerol) in the presence of protease (Sigma-Aldrich, Saint Louis, MO, USA) and phosphatase (Roche, Basel, Switzerland) inhibitor cocktails and homogenized at 4 °C by scraping. Proteins in the supernatant were separated on a 10% sodium dodecyl sulfate (SDS)-polyacrylamide gel and electrotransferred to polyvinylidene difluoride (PVDF) membranes. The following primary antibodies were used for the Western blot: anti-cGKIα (1:400, Santa Cruz Biotechnology, Dallas, TX, USA, catalog no. sc-10338), anti-AMPKα (1:1000, Cell Signaling Technology, Danvers, MA, USA, catalog no. 2532L), anti-AMPKα1 (1:500, Santa Cruz Biotechnology, Dallas, TX, USA, catalog no. sc-19128), anti-AMPKα2 (1:500, Santa Cruz Biotechnology, Dallas, TX, USA, catalog no. sc-19129), anti-p-AMPKα (Thr172) (1:1000, Cell Signaling Technology, Danvers, MA, USA, catalog no. 2535L), anti-p-MYPT1 (Ser695) (1:400, Santa Cruz Biotechnology, Dallas, TX, USA, catalog no. sc-33360), anti-p-MLC2 (Ser19) (1:400, Cell Signaling Technology, Danvers, MA, USA, catalog no. 3671L), and anti-actin (1:16000, Sigma-Aldrich, Saint Louis, MO, USA, catalog no. A3853). To detect the primary antibodies, the membranes were incubated with appropriate alkaline phosphatase-labeled secondary antibodies (Sigma-Aldrich, Saint Louis, MO, USA). The protein bands were visualized using the colorimetric 5-bromo-4-chloro-3-indolylphasphate/nitroblue tetrazolium system. The densitometric quantification of bands was performed using Quantity One 4.6.6 software (Bio-Rad Laboratories, Hercules, CA, USA).

### 4.3. Immunoprecipitation

Podocyte extracts were precleared with mouse IgG plus Protein A/G-PLUS Agarose at 4 °C for 1 h and then incubated with an appropriate primary antibody plus Protein A/G-PLUS Agarose at 4 °C overnight. The agarose beads were washed gently with lysis buffer. Proteins were then eluted from the beads by adding SDS loading buffer. Afterward, the sample was boiled for 5 min and analyzed by Western blot.

### 4.4. siRNA Transfection

Podocytes were transfected with siRNA that targeted PKGIα, AMPKα1, or AMPKα2 or non-silencing siRNA (scrambled siRNA, negative control; Santa Cruz Biotechnology, Dallas, TX, USA). Cells were cultured in RPMI-1640 medium that was supplemented with 10% fetal bovine serum (FBS). One day before the experiment, the culture medium was changed to antibiotic-free RPMI-1640, which was supplemented with 10% FBS. The cells were transfected with siRNAs using siRNA Transfection Reagent (OriGene, Rockville, MD, USA) according to the manufacturer’s instructions. Briefly, the targeted siRNA or scrambled siRNA was diluted in transfection medium (final concentration, 80 nM), mixed with siRNA transfection reagent, and incubated for 30 min at room temperature. The transfection medium was then added to the transfection mixture, mixed gently, and added to the podocytes. After 7 h, growth medium that was supplemented with 2× higher concentrations of FBS and antibiotics was added to the cells. Afterward, the podocytes were incubated for an additional 24 h. After transfection, gene-silencing was checked at the protein level by Western blot.

### 4.5. Immunofluorescence

Podocytes were seeded on coverslips that were coated with type I collagen (Becton Dickinson Labware, Becton, UK) and cultured in RPMI-1640 medium that was supplemented with 10% FBS. Cells were fixed in phosphate-buffered saline (PBS) plus 4% formaldehyde for 20 min at room temperature. Fixed podocytes were permeabilized with 0.1% Triton-X for 3 min and then blocked with PBSB solution (PBS plus 2% FBS, 2% bovine serum albumin (BSA), and 0.2% fish gelatin) for 1 h. After blocking, the cells were incubated with anti-AMPKα1 (1:100), anti-AMPKα2 (1:100), and anti-PKGIα (1:15) antibodies in PBSB at 4 °C for 1.5 h. The primary antibodies were incubated with a blocking peptide to eliminate nonspecific staining. Next, the cells were washed three times with cold PBS and incubated with secondary antibodies that were conjugated to Alexa Fluor 488 (1:750) or Alexa Fluor 546 (1:750). Specimens were imaged using a confocal laser scanning microscope (Leica SP8X, Wetzlar, Germany) with a 63× oil immersion lens. Actin was stained using Alexa Fluor 633 phalloidin (1:200) and imaged using a Nikon Ti Eclipse confocal laser scanning microscope (Nikon Instruments Inc., Minato, Tokyo, Japan) with a 40× lens.

### 4.6. Permeability Assay

The transepithelial permeability to albumin was investigated by measuring the diffusion of FITC-labeled BSA (Sigma-Aldrich, Saint Louis, MO, USA, catalog no. A9771) across the podocyte monolayer as described previously [11,52]. Briefly, podocytes (25 × 10^3^ cells/well) were seeded on 3-μm membrane pore size cell culture inserts that were coated with type IV collagen (Corning, NY, USA) and placed in 24-well plates. Transwell permeability experiments were conducted on differentiated cells between 7 and 15 days post-seeding. Before the experiments, podocytes were washed twice with PBS, and the medium on both sides of the insert was replaced with serum-free RPMI-1640 medium (SFM). After 2 h, the medium in the upper compartment was replaced with 0.3 mL of fresh SFM, and the medium in the lower compartment was replaced with 1.3 mL of SFM that was supplemented with 1 mg/mL FITC-albumin. After 1 h of incubation, 150 μL of the solution from the upper chamber was transferred to a 96-well plate, and the absorbance of FITC-albumin was measured at 490 nm using an EL808 Absorbance Reader (BioTek Instruments, Winooski, VT, USA).

Albumin concentrations were calculated based on standard concentrations that were prepared in SFM, ranging from 0.01 to 0.5 mg/mL FITC-albumin. The emission signals from SFM were subtracted from the standards and FITC-albumin samples. The linear calibration curve was plotted, with the standard concentration on the x-axis and optical density values on the y-axis. Based on the calibration curve, the equation of the straight line that fits the standard concentration data was generated. The FITC-albumin concentrations were calculated based on the linear function *y = ax + b*, where *y* is the optical density value, *a* is the slope of the line, *x* is the unknown FITC-albumin concentration, and *b* is the y intercept. The variation in FITC-albumin concentration between separate experiments may be the result of using inserts from different manufacturers. We previously used BioCoat Control Inserts with 3.0 µm PET Membrane (catalog no. 354575). However, this product was discontinued, and Transwell-COL Permeable Supports with a 3.0 µm PTFE Membrane (Costar, catalog no. 3496, Corning, NY, USA) were used instead. The membranes are made of different materials that may affect permeability to FITC-albumin in some way.

### 4.7. Isolation of Rat Glomeruli

Kidneys from 6 week old male Wistar rats were removed and placed in supplemented ice-cold PBS (pH 7.4; 137 mM NaCl, 2.7 mM KCl, 8.1 mM Na_2_HPO_4_, 1.5 mM KH_2_PO_4_, 0.49 mM MgCl_2_, 0.9 mM CaCl_2_, and 5.6 mM glucose). Next, the renal capsule was removed, and the cortex was minced with a razor blade and then pressed through a system of sieves with decreasing pore diameters (250, 125, and 75 μm). The obtained cell suspension contained decapsulated glomeruli without afferent and efferent arterioles. The entire procedure was performed in an ice bath and completed in less than 1 h.

### 4.8. Glomerular Permeability to Albumin In Vitro

The permeability of the glomerular capillary wall in response to an oncotic gradient that was generated by changes in the determined concentration of albumin in the experimental medium was measured as described previously [53] with slight modifications. Isolated glomeruli were affixed to 0.1% poly-L-lysine-coated plates for 10 min. Unattached glomeruli were removed by gently washing with fresh 5% BSA in supplemented PBS. Subsequently, the glomeruli were incubated for an additional 5 min, and the volume responses of glomeruli to changes in albumin concentration were recorded. Subsequently, glomeruli that were incubated in 5% BSA medium were treated with an AMPK inhibitor (100 μM compound C, 20 min) or AMPK activators (MTF, 2 mM, and 30 min; AICAR, 100 μM and 20 min) and a PKG activator (100 μM 8-Br-cGMP, 5 min) or PKG inhibitor (100 μM Rp-8-Br-cGMPS, 20 min) at 37 °C. Next, the compounds were washed twice with 5% BSA medium. The 5% BSA medium was then replaced with 1% BSA medium to generate an oncotic gradient across the glomerular capillary wall. Control glomeruli were treated with equivalent volumes of 5% BSA medium that did not generate an oncotic gradient. Changes in glomerular volume were recorded by videomicroscopy (Olympus IX51 microscope, Olympus Corporation, Tokyo, Japan) before the activity modulators were added and 1 min after they were added. Glomerular volume (V) was calculated based on the surface area (S) of the glomerulus according to the following formula using CellSens Dimension 1.18 software (Olympus Corporation, Tokyo, Japan): V = $\left[\frac{4}{3}\times S\left(\sqrt{S}/\pi \right)\right]/10^{6}$. There is a direct relationship between the increase in glomerular volume (ΔV), calculated as (V_final_ − V_initial_)/V_initial_, and the oncotic gradient (ΔΠ) that is applied across the capillary wall. This principle was used to calculate the reflection coefficient of albumin (σ_alb_), defined as the ratio of ΔV that is measured in the presence (experimental) and absence (control) of an oncotic gradient: σ_alb_ = ΔV_experimental_/ΔV_control_. The σ_alb_ value was then used to calculate glomerular capillary permeability to albumin (P_alb_), expressed as P_alb_ = 1 − σ_alb_, which describes the albumin current flow consequent to water flow. To obtain reliable results and preserve glomerular viability during the experiment, the glomerular permeability assay was performed for no more than 1 h; therefore, the glomeruli were incubated with compounds for ≤30 min. At least 12–16 glomeruli that were isolated from four rats were studied.

### 4.9. Extraction of Nucleotides and High-Performance Liquid Chromatography

The extraction of nucleotides from cells was performed by modifying the procedure of Smolenski et al. [54]. Podocytes were differentiated and cultured on six-well plates. On the day of the experiment, the cells were washed with PBS, and 0.5 mL of cold 0.4 M HClO_4_ was added to each well. The plate was frozen at −80 °C for 24 h. Afterward, the cells were thawed on ice, collected in Eppendorf tubes, and centrifuged at 14,000 rotations per minute for 10 min at 4 °C. The supernatants were adjusted to a neutral pH with 2 M K_2_HPO_4_, centrifuged, filtered with 0.2 μm RC-membranes (Minisart RC4, Sartorius, UK), and analyzed by high-performance liquid chromatography (HPLC) with a UV-Vis detector.

Nucleotides were quantified using a Perkin Elmer Series 200 that consisted of a chromatographic interface (Link 600), binary pump, UV-Vis detector, and vacuum degasser. A Gemini 5 μm C18 110 Å 150 × 4.6 mm column, protected by a Gemini C18 4 × 3 mm guard column (Phenomenex, Torrance, CA, USA), was used for chromatographic separation. All compounds were detected at a wavelength of 254 nm. The mobile phase was adapted from a previous study [55] and consisted of a 50 mM phosphate buffer with 4 mM tetrabutylammonium hydrogen sulfate, adjusted to pH 6 with orthophosphoric acid (solution A) and HPLC-grade acetonitrile (solution B). The flow rate was 1 mL/min, and the applied gradient was the following: 0–5 min (95% solution A and 5% solution B), 5-12 min (amount of solution B increased linearly to 15%), 12–15 min (85% solution A, 15% solution B), and 15–17 min (gradient returned linearly to initial conditions of 95% solution A and 5% solution B). The runtime for the elution of nucleotides was 17 min. The column was equilibrated between injections for 20 min. The injection volume was set to 100 μL.

### 4.10. Statistical Analysis

All statistical analyses were performed using GraphPad Prism 8 software. The Shapiro–Wilk test was used to determine a normal distribution of datasets. Depending on the result of the normality test, the data were analyzed using a parametric test (analysis of variance followed by Tukey’s, Sidak’s, or Dunnett’s multiple-comparison post hoc test or unpaired *t-*test) or nonparametric test (Kruskal–Wallis test followed by Dunn’s multiple-comparison post hoc test) to determine significance. The data are expressed as means ± SEM. Values of *p* < 0.05 were considered statistically significant.

## 5. Conclusions

The interplay between PKGIα and AMPKα2 appears to be an important regulatory mechanism of podocytes, which maintains the proper function of the GFB. In pathological states, such as insulin resistance, diabetes, and hyperglycemia, balanced interactions between PKGIα and AMPKα activity might be impaired in podocytes, leading to PKGIα overactivity and an increase in the permeability of the GFB. The newly discovered crosstalk between PKGIα and AMPKα2 broadens our knowledge of the physiology of podocytes and suggests a new mechanism that may be disturbed in diabetes, leading to podocyte dysfunction. Understanding the mechanism of regulation of the PKGIα and AMPKα2 interaction at the molecular level provides further insights into glomerular disease pathogenesis and novel therapeutic targets for glomerulopathies.

## Data Availability

Data that are presented in this study are available upon request from the corresponding author.
